# Simultaneous Determination of Seven Anthraquinone Aglycones of Crude and Processed Semen Cassiae Extracts in Rat Plasma by UPLC–MS/MS and Its Application to a Comparative Pharmacokinetic Study

**DOI:** 10.3390/molecules22111803

**Published:** 2017-10-28

**Authors:** Rixin Guo, Hongwei Wu, Xiankuo Yu, Mengying Xu, Xiao Zhang, Liying Tang, Zhuju Wang

**Affiliations:** 1 Institute of Chinese Materia Medica, China Academy of Chinese Medical Science, No. 16 Nanxiaojie, Dongzhimennei Ave., Beijing 100700, China; guorixinxt@163.com (R.G.); hwwu@icmm.ac.cn (H.W.); yuxiankuobz@126.com (X.Y.); 2 School of Medicine, Henan University of Chinese Medicine, No. 156 Jinshuidong Ave., Zhengzhou 450046, China; 13501288046@163.com (M.X.); 13633838012@163.com (X.Z.)

**Keywords:** UPLC-ESI-MS/MS, Semen cassiae, anthraquinone aglycone, pharmacokinetics

## Abstract

Semen cassiae is the ripe seed of *Cassia obtusifolia* L. or *Cassia tora* L. of the family Leguminosae. In traditional Chinese medicine, the two forms of Semen cassiae are raw Semen cassiae (R-SC) and parched Semen cassiae (P-SC). To clarify the processing mechanism of Semen cassiae, the pharmacokinetics of R-SC and P-SC extracts were examined. A simple, rapid, sensitive ultra-high-performance liquid chromatography–tandem mass spectroscopy (UPLC-MS/MS) method was developed and validated for the simultaneous determination of seven anthraquinone aglycones of Semen cassiae (aurantio-obtusin, obtusifolin, questin, 2-hydroxyemodin-1-methyl-ether, rhein, emodin, 1,2,7-trimethoxyl-6,8-dihydroxy-3-methylanthraquinone) to compare the pharmacokinetics of raw and parched Semen cassiae in rat plasma. Compared with the R-SC group, C_max_ and AUC_0-12_ tended to be higher in the P-SC group. In particular, C_max_ values for aurantio-obtusin, obtusifolin, questin, 2-hydroxyemodin-1-methyl-ether and rhein were significantly higher in the P-SC group (*p* < 0.05). Meanwhile, T_max_ and MRT_0-12_ tended to be lower in the P-SC group. Specifically, T_max_ for aurantio-obtusin and 2-hydroxyemodin-1-methyl-ether and MRT_0-12_ for obtusifolin and rhein were significantly higher in the P-SC group (*p* < 0.05).

## 1. Introduction

As most Chinese medicinal herbs are obtained from crude plants, some are mixed with impurities in the collecting process, some are violently toxic, thus precluding their direct consumption, whereas others cannot produce the intended therapeutic effect without special treatment. Thus, they must be subjected to processing, such as parching, steaming and boiling, before clinical use. These processing methods are called ‘Pao Zhi’ in Chinese. The acknowledged theory of the seeds of herbal medicine used in traditional Chinese medicine is summarized as ‘Feng zi bi chao’, meaning that they must be parched or fried before clinical use. Why is parching necessary? The theoretical basis was discovered during the Ming Dynasty in Yizong Cuiyan [1]. Ancient people believed that parching or frying the seeds could increase the levels of active compounds in the decoction. After a long period of evolution and transfer, seed herb medicinal processing rules have been developed. Although the purpose of parching or frying is not identical for every single seed, the increase in the dissolution rate of the effective components is the same.

Semen cassiae is the ripe seed of *Cassia obtusifolia* L. or *Cassia tora* L. of the family Leguminosae. It was initially recorded in Shennong Bencao Jing [2], which is the earliest book of Chinese materia medica, during the Han Dynasty of China. In this monograph, Semen cassiae was described for use as a laxative and for reversing ‘‘liver fire’’ to improve vision. In addition, it has been widely used in Korea, Japan, India and many Southeast Asian countries as an herbal medicine or health tea for the treatment of a variety of ailments [3,4,5]. Modern pharmacological studies indicate that Semen cassiae has hepatoprotective activities [6], antioxidants activities [7,8], neuroprotective effects [9,10,11] and antidiabetic effects [12]. In the clinical practice of Chinese medicine, Semen cassiae is used as raw Semen cassiae (R-SC) and parched Semen cassiae (P-SC), both of which are recorded in Chinese Pharmacopoeia. P-SC, which was first described in Taiping Shenghui Fang [13] during the Song Dynasty of China, has always been preferred by doctors. In addition, P-SC is generally associated with decreased purgative effects, making it suitable for elderly and infirm patients [14]. Modern pharmacological studies also indicated that P-SC has decreased purgative effects compared with R-SC [15]. A chemistry study identified anthraquinone aglycones, anthraquinone glycosides and naphthopyrone glycosides as the main constituents of Semen cassiae [16,17]. Changes of the clinical curative effect are assumed to be related to changes of the internal chemical composition, including qualitative and quantitative changes. In our previous study, we discovered that parching can increase the contents of three anthraquinone aglycones (aurantio-obtusin, chryso-obtusin, obtusifolin) and decrease the contents of three naphthopyrone glycosides (rubrofusarin gentiobioside, casside, cassiaside C) [18]. However, the active compounds responsible for the therapeutic effects were generally the absorbed ingredients in vivo. Bioactive herbal compounds that exhibit considerable levels after administration are most likely to account for the pharmacological effects of herbal medicines and form the basis of therapeutic efficacy. Thus, studying the chemical composition of R-SC or P-SC is not sufficient to reveal the processing mechanism. Pharmacokinetic studies of R-SC and P-SC extract should be performed to further clarify the processing mechanism of Semen cassiae.

With its high selective and rapid analysis properties, ultra-high-performance liquid chromatography–tandem mass spectroscopy (UPLC–MS/MS) is widely used in pharmacokinetic analyses of many medicines. After oral administration of herbal medicines, the components in blood are extremely complex, and their concentrations are extremely low. Thus, UPLC–MS/MS had outstanding advantages for pharmacokinetic studies of natural medicines. Concerning the pharmacokinetic analysis of oral R-SC, UPLC–MS/MS exhibited feasibility for detecting some compounds of Semen cassiae such as aurantio-obtusin, chryso-obtusin and obtusin in the reported literature [19,20]. However, no studies of the pharmacokinetics of P-SC have been reported.

**Figure 1:**
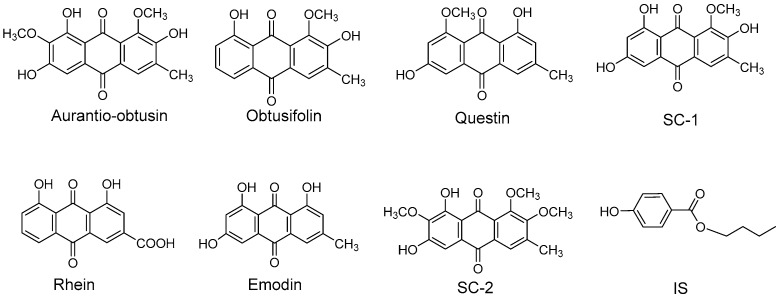
Chemical structures of seven analytes and internal standard.

In this study, seven anthraquinone aglycones, namely aurantio-obtusin, obtusifolin, questin, 2-hydroxyemodin-1-methyl-ether (SC-1), rhein, emodin and 1,2,7-trimethoxyl-6,8-dihydroxy-3-methylanthraquinone (SC-2), were selected as quantitative components in plasma. All chemical structures are shown in Figure 1. Next, a selective and sensitive UPLC–electrospray ionisation (ESI)–MS/MS method was developed for the simultaneous quantification of the seven compounds in rat plasma. The established method was then successfully applied to a pharmacokinetic study in male rats after the oral administration of R-SC and P-SC extracts.

For pharmacokinetic analyses, blood is usually collected from the orbital venous plexus, but this method results in the waste of blood and pain to the animals, including death. We used an automated blood sampler to collect blood from the rats. By programming the sample time and volumes, the exact peristaltic pump of the sampler will automatically collect blood samples from the jugular vein of rats via a catheter. The automated blood sampler increases the precision of sampling, avoids leakage and reduces wasting of blood samples. Anaesthesia is not needed, thus eliminating the effects of anaesthesia on pharmacokinetics. It also reduces the discomfort of animals during the experiment.

## 2. Results

### 2.1. Method Validation

#### 2.1.1. Specificity

**Figure 2:**
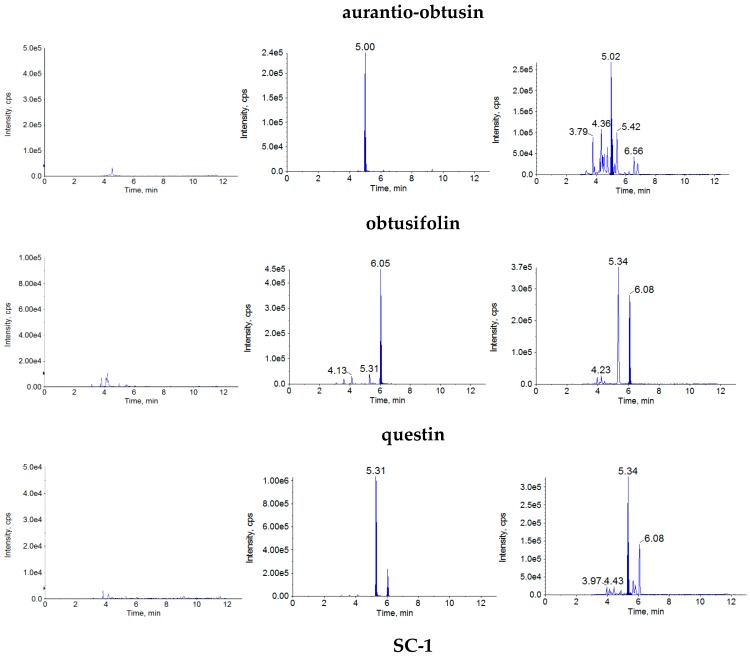

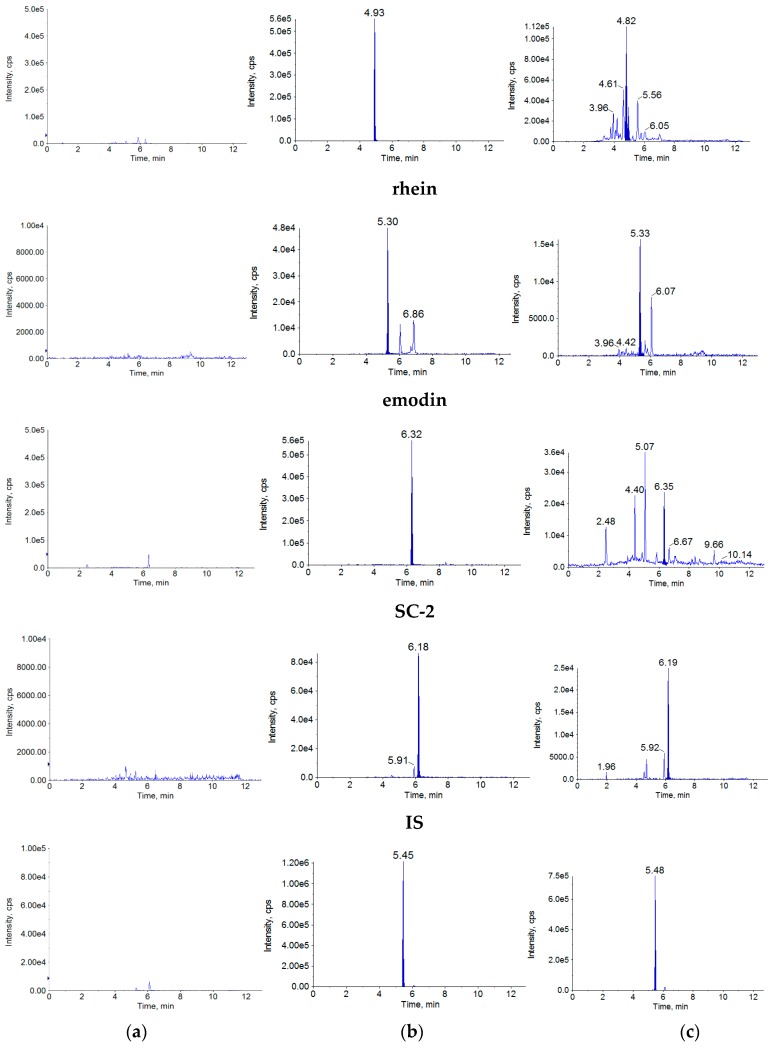
MRM chromatograms of the analytes in rat plasma: blank plasma (**a**), blank rat plasma spiked with the analytes and IS (**b**) and plasma samples taken from rats at 30 min after oral administration of R-SC extract (**c**).

The retention times were approximately 5.04 min for aurantio-obtusin, 6.09 min for obtusifolin, 5.34 min for questin, 4.96 min for SC-1, 5.34 min for rhein, 6.37 min for emodin, 6.22 min for SC-2 and 5.46 min for internal standard (IS). There was no obvious interference by endogenous substances (Figure 2).

#### 2.1.2. Linearity and LLOQ

The final concentrations of the calibration samples were in the ranges of 0.94–564.0 ng/mL for aurantio-obtusin, 1.03–410.0 ng/mL for obtusifolin, 0.84–84 ng/mL for questin, 1.03–82.80 ng/mL for SC-1, 1.07–203.0 ng/mL for rhein, 1.03–207.0 ng/mL for emodin and 0.9–180.0 ng/mL for SC-2. In the calibration equations, X is the plasma concentration of each analyte (ng/mL), Y is the peak-area ratio of each analyte to IS, and r is the correlation coefficient. All seven calibration curves exhibited good linearity for their corresponding analytes (r > 0.9964). The LLOQ values were 0.94 ng/mL for aurantio-obtusin, 1.03 ng/mL for obtusifolin, 0.84 ng/mL for questin, 1.03 ng/mL for SC-1, 1.07 ng/mL for rhein, 1.03 ng/mL for emodin and 0.90 ng/mL for SC-2. The results for the calibration curves, correlation coefficients and LLOQ of the analytes in rat plasma are shown in Table 1.

#### 2.1.3. Precision and Accuracy

The intra- and inter-day precisions and accuracies for the plasma samples are shown in Table 2. For each QC sample, the intra- and inter-day precision values (RSD, %) were both less than 9%, and the accuracy (%) was in the range of 95–106%. These results demonstrated that the precision and accuracy were all within acceptable ranges, and this method proved to be accurate and precise.

#### 2.1.4. Recovery and Matrix Effect

The recoveries and matrix effects of the seven analytes at three concentrations are shown in Table 3. The recoveries of the analytes ranged 76–94%, and the matrix effects ranged 93–106%. The results indicated acceptable recoveries and matrix effects for the present method.

#### 2.1.5. Stability Experiments

The stability data indicated that the seven analytes were all stable in plasma for all analyzed conditions, as shown in Table 4.

### 2.2. Application to the Pharmacokinetic Study

The concentrations of the seven analytes in the R-SC and P-SC extracts were determined by UPLC–MS/MS. The results illustrated that the concentrations of the analytes in the R-SC and P-SC extracts were 578 and 918 μg/mL, respectively, for aurantio-obtusin, 178 and 352 μg/mL, respectively, for obtusifolin, 28 and 40 μg/mL, respectively, for questin, 96 and 163 μg/mL, respectively, for SC-1, 26 and 40 μg/mL, respectively, for rhein, 74 and 117 μg/mL, respectively, for emodin and 44 and 76 μg/mL, respectively, for SC-2.

**Figure 3:**
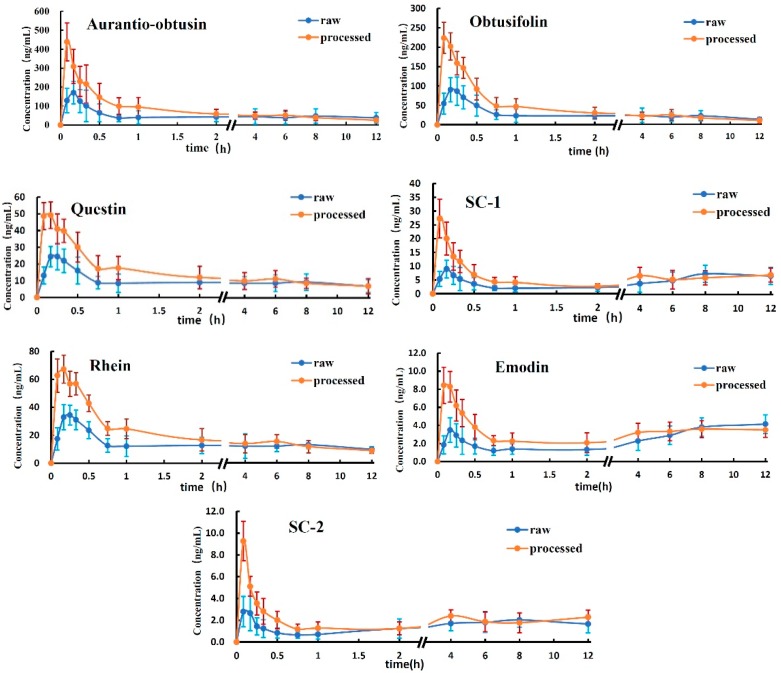
Plasma concentration-time of the analytes in rats given single oral administration.

In this study, the validated analytical method was employed to determine the concentrations of the seven analytes in rat plasma after oral administration (gavage) of the R-SC and P-SC extracts. The mean plasma concentration–time profiles of the seven analytes are presented in Figure 3. The pharmacokinetic parameters, including C_max_, T_max_, AUC_0-12_, AUC_0-∞_ and MRT_0-12_ were calculated.

As shown in Table 5, C_max_ was larger for all seven analytes in the P-SC group, with significant differences observed for aurantio-obtusin, obtusifolin, questin, SC-1 and rhein (*p* < 0.05).

Regarding AUC, there were no significant differences in AUC_0-∞_ and AUC_0-12_ for the seven analytes between the P-SC and R-SC group. However, AUC_0-12_ was higher for all seven analytes in the P-SC group than in the R-SC group.

The mean T_max_ and MRT_0-12_ values of the analytes were all smaller in the P-SC group than in the R-SC group. Specifically, T_max_ for aurantio-obtusin and SC-1 and MRT_0-12_ for obtusifolin and rhein were significantly smaller in the P-SC group (*p* < 0.05).

## 3. Discussion

### 3.1. Dosage of the Seven Analytes

In the first step of a pharmacokinetic analysis of an herbal medicine, except in cases in which the components of the medicine exhibit considerable systemic levels after dosing, another important question concerns the exact dosage for the analyzed herbal compounds. In this study, although the dosages of R-SC and P-SC calculated using the crude drug were identical, the exact dosages of the seven analytes in the extracts were different.

The dosages of these analytes in the R-SC and P-SC extracts were showed in Table 6. As the concentrations of the seven analytes were higher in the P-SC extract than in the R-SC extract, their initial dosages were also higher.

### 3.2. Analysis of Analytes Concentration in the Plasma

C_max_ is often measured to demonstrate bioequivalence. According to the FDA, bioequivalence and bioavailability often rely on pharmacokinetic measurements such as AUC and C_max_ that are reflective of systemic exposure.

As shown in Table 5, the mean C_max_ values of the seven analytes were higher in the P-SC group than in the R-SC group. Via a calculation based on the results in Section 3.1, the ratios of the dosages of the aurantio-obtusin, obtusifolin, questin, SC-1, rhein, emodin and SC-2 between P-SC and R-SC were 1.6, 2.0, 1.5, 1.7, 1.6, 1.5 and 1.7, respectively, and the ratios of the mean C_max_ values of the analytes between P-SC and R-SC were 2.6, 2.3, 2.0, 2.5, 1.9, 2.0 and 2.4, respectively. Thus, the differences in the mean C_max_ were obviously larger than the differences in the dosages. The results implied that the parching process increased both the contents of the seven analytes in Semen cassiae and the rate of absorption.

As shown in Figure 3, 2 h after oral administration (all T_max_ values were within 15 min), the concentrations of some anthraquinone aglycones tended to increase again in the concentration–time curve in the R-SC group. In particular, SC-1 and emodin levels in plasma appeared another top level at 8 h in the R-SC group. This pharmacokinetic characteristic of some anthraquinone aglycones after oral administration was coincident with previous findings [19,20,21]. The reason may be that during anthraquinone aglycone absorption, more anthraquinone aglycones could be produced from the corresponding anthraquinone glycosides by intestinal bacteria. The newly produced aglycones were absorbed into blood, causing an additional increase in absorption in the concentration–time curve. Compared with the data for P-SC, the anthraquinone glycoside contents in R-SC were higher. This change was more obvious in the R-SC group.

### 3.3. Analysis of the Mean AUC

AUC is typically used to reflect bioavailability and bioequivalence. As shown in Table 5, there were no significant differences in AUC_0-∞_ and AUC_0-12_ for the seven analytes between the P-SC and R-SC groups. However, AUC_0-12_ tended to be higher for all seven analytes in the P-SC group. Furthermore, from the plasma concentration vs. time curve (Figure 3), it was clear that within 2 h after oral administration, the curves of the seven analytes were obviously larger in the P-SC group (orange) than in the R-SC curves (blue). The results suggested that with the same dosage calculated using the crude drug within 2 h after oral administration, the bioavailability of the seven analytes was higher in P-SC than in R-SC.

### 3.4. Analysis of T_max_ and MRT_0-12_

T_max_ was within 15 min after oral administration for all seven analytes, suggesting that the seven anthraquinone aglycones were rapidly absorbed into blood. The mean T_max_ values of the analytes were smaller in the P-SC group than in the R-SC group (Table 5). In particular, the T_max_ values for aurantio-obtusin and SC-1 were significantly smaller in the P-SC group (*p* < 0.05). These results suggested that for Semen cassiae, processing can accelerate the absorption of aglycones, causing the blood concentration to peak more rapidly. Similar results were obtained for MRT. As shown in Table 5, the mean MRT_0-12_ values of the analytes were all smaller in the P-SC group than in the R-SC group, with significant differences noted for obtusifolin and rhein.

## 4. Materials and Methods

### 4.1. Chemicals and Reagents

Aurantio-obtusin (No. 111900-201303, ≥99.1%) and rhein (No. 110757-200206, ≥99.3%) were purchased from the National Institutes for Food and Drug Control (Beijing, China). Obtusifolin, questin, SC-1, emodin were prepared in our laboratory [22], and their structures were determined via comparisons of spectral data (UV, MS, ^1^H-NMR and ^13^C-NMR) with values reported in the literature. SC-2 was first isolated in our laboratory [23]. The purity of these compounds was determined to be ≥98% by HPLC using the normalization method of peak area. Butyl 4-hydroxybenzoate ([IS], ≥98%, No: CHB-N-025) was purchased from Chengdu Chroma-Biotechnology Co., Ltd. (Chengdu, China). All chemicals were stored at 4 °C in our laboratory. Acetonitrile, methanol, water and formic acid (Fisher, Fair Lawn, NJ, USA) were of UPLC grade.

### 4.2. Instrument and Analytical Conditions

The UPLC–MS/MS system consisted of a Shimadzu LC-20AD series liquid chromatograph (Shimadzu, Kyoto, Japan) and a 5500 triple quadrupole mass spectrometer (Applied Bio-systems, AB Sciex, Framingham, MA, USA) equipped with an ESI source system. Data acquisition was performed using Analyst 1.6.2 software (AB MDS Sciex, Framingham, MA, USA). The automated blood sampler (Instech, Plymouth, PA, USA) was equipped with Instech Automated Blood Sampling System ABS212 Software Version 2.13.1 (Instech, Plymouth, PA, USA).

The separations were achieved on a Waters HSS T3 column (100 mm × 2.1 mm, 1.7 μm, Waters Corporation, Milford, MA, USA). The mobile phases consisted of 0.1% formic acid (A) and acetonitrile (B) using a gradient elution as follows: 90% to 75% A and 10% to 25% B (*v*/*v*) at 0–1 min; 75% to 30% A and 25% to 70% B (*v*/*v*) at 1–3 min; 30% to 10% A and 70% to 90% B (*v*/*v*) at 3–10 min; 10% to 90% A and 90% to 10% B (*v*/*v*) at 10–10.1 min; and 90% A and 10% B (*v*/*v*) at 10.1–13 min. The flow rate was set at 0.3 mL/min. The column oven temperature was kept at 40 °C. The injection volume was 1.0 μL. The full-scan mass spectra revealed that ionisation of the seven analytes was more efficient in the negative-ion mode than in positive-ion mode; thus, quantification was performed in the negative-ion mode. Quantification was performed using multiple reaction monitoring of the precursor product ion transition at *m*/*z* 329.1–314.0 for aurantio-obtusin, *m*/*z* 283.1–268.0 for obtusifolin, *m*/*z* 283.1–240.0 for questin, *m*/*z* 299.1–256.0 for SC-1, *m*/*z* 283.0–183.0 for rhein, *m*/*z* 269.0–225.1 for emodin, *m*/*z* 343.1–328.1 for SC-2 and *m*/*z* 193.0–136.0 for butyl 4-hydroxybenzoate. The other parameters of the mass spectrometer were as follows: curtain gas, 30.00 psi; collision gas, −2 psi; ion spray voltage, −4500.00 V; temperature, 550.00 °C; ion source gas 1, 55.00 psi; and ion source gas 2, 55.00 psi. The dwell time of each MRM transition was 300 ms.

### 4.3. Preparation of the R-SC and P-SC Extracts

R-SC was purchased from Bozhou TCM market (Anhui, China) and authenticated by Professor Zhuju Wang. R-SC was processed according to the procedure recorded in Chinese Pharmacopoeia to obtain P-SC (National Commission of Chinese Pharmacopoeia, 2015). Briefly, after heating a pan to 200 °C, R-SC was placed in the pan and constantly stirred for 5 min, after which the seeds were removed for cooling. The content determination standard of Chinese Pharmacopoeia (2015 edition) was achieved for both R-SC and P-SC. The voucher specimens (voucher no. R-SC 160708, P-SC 160905) are deposited at the herbarium of China Academy of Chinese Medical Science (Beijing, China).

To prepare the R-SC and P-SC extracts, 50 g of R-SC (or P-SC) dried powder were extracted under reflux with 500 mL of 75% methanol for 1 h and then filtrated. Approximately 5 mL of the filtrate were stored at 4 °C for quantitative analysis. Then, 415 mL of the filtrate were evaporated to dryness, and the dense extract was dissolved in water to obtain 23 mL of the R-SC (or P-SC) extract for oral administration.

### 4.4. Preparation of Combined Standard Stock Solution and IS Solution

The combined standard stock solution of aurantio-obtusin (11.3 μg/mL), obtusifolin (12.3 μg/mL), questin (10.1 μg/mL), SC-1 (12.4 μg/mL), rhein (12.8 μg/mL), emodin (12.4 μg/mL) and SC-2 (10.1 μg/mL) was prepared in methanol, as was the IS solution (104 ng/mL). All solutions were stored at 4 °C.

### 4.5. Preparation of Calibration Standard and Quality Control (QC) Samples

The combined standard stock solution was further diluted in methanol to produce a series of standard working solutions. Calibration standards were prepared by spiking 100 μL of the standard working solutions into 100 μL blank plasma to yield calibration concentration of 0.94–564.0 ng/mL for aurantio-obtusin, 1.03–410.0 ng/mL for obtusifolin, 0.84–84.0 ng/mL for questin, 1.03–82.80 ng/mL for SC-1, 1.07–203.0 ng/mL for rhein, 1.03–207.0 ng/mL for emodin, 0.90–180.0 ng/mL for SC-2. The QC samples were prepared in blank plasma at three concentrations (low, medium and high), which were 1.88, 18.8 and 94 ng/mL for aurantio-obtusin; 2.05, 20.5 and 103 ng/mL for obtusifolin; 1.68, 16.8 and 84 ng/mL for questin; 2.07, 20.7 and 103 ng/mL for SC-1; 2.13, 21.3 and 107 ng/mL for rhein; 2.07, 20.7 and 207 ng/mL for emodin and 1.8, 18.0 and 90 ng/mL for SC-2.

### 4.6. Plasma Samples Preparation

To a 100-μL aliquot of plasma, 100 μL of the IS working solution were added and vortexed for 30 s. Next, 400 μL of acetonitrile were added, vortexed for 1 min and centrifuged for 10 min at 10,000 rpm. Then, the supernatant were transferred to a 1.5-mL centrifuge tube and evaporated to dryness under a stream of nitrogen gas at 40 °C. The dried residue was reconstituted in 100 μL of methanol, vortexed for 1 min and centrifuged at 10,000 rpm for 10 min. Then, a 1.0-μL aliquot of each sample was injected into the UPLC–MS/MS system for calibration analysis.

### 4.7. Method Validation

The method was validated for specificity, linearity, lower limit of quantification (LLOQ), accuracy and precision, recovery, matrix effect and stability in accordance with the requirements of the bioanalytical method validation guidelines of the USA Food and Drug Administration (FDA) and registration guidelines for new drugs in China.

#### 4.7.1. Specificity

The specificity of this method was evaluated by comparing the chromatograms of the blank plasma samples from six individual rats, blank plasma spiked with the seven analytes and IS and plasma samples obtained from the rats after the oral administration of the R-SC and P-SC extracts.

#### 4.7.2. Linearity and LLOQ

Calibration samples were injected into the UPLC–MS/MS system for calibration analysis. The calibration curves were constructed by plotting the ratio of the chromatographic peaks area (analytes/IS) versus the concentration of these analytes using the least-squares linear regression method to calculate the calibration equation and regression parameters. LLOQ was defined as the lowest concentration on the calibration curve with acceptable precision and accuracy.

#### 4.7.3. Precision and Accuracy

Precision and accuracy were assessed via replicate analysis (*n* = 6) of QC samples at high, medium and low concentrations on the same day (intra-day) and on 3 different days (inter-day). The actual concentration of each QC sample was obtained using calibration curves prepared the same day. The intra-day and inter-day precisions were expressed as the relative standard deviation (RSD), and the accuracy was defined by comparing the measured concentration using calibration curves with its nominal value.

#### 4.7.4. Recovery and Matrix Effect

To determine recovery and the matrix effect, processed, non-processed (pure sample freshly prepared in methanol) and post-processed spiked samples at the three QC concentrations were analysed. Recovery was determined at the three QC concentrations with six replicates by comparing the observed peak areas of processed plasma samples with those of non-processed samples. The matrix effect was evaluated by comparing the peak areas of the analytes in post-processed spiked samples with those of the analytes in non-processed samples at the same theoretical concentrations.

#### 4.7.5. Stability Experiments

The stability of analytes in plasma was assessed by analysing QC samples (high, medium and low concentrations) under following conditions: room temperature for 4 h, autosampler for 24 h, three freeze/thaw cycles (from −20 °C to ambient temperature) and long-term stability (storage at −20 °C for 3 weeks). Stability was expressed as percentage of the remaining concentration.

### 4.8. Application of Method and Pharmacokinetic Study

Male Sprague–Dawley rats (body weight, 310 ± 20 g) were purchased from the Peking University Health Science Center (Beijing, China) and adapted under 65% relative humidity at 25 °C for 5 days. The animal handling procedures were approved by the Institutional ethical committee China Academy of Chinese Medical Science (approval No.: 2016090110). The animals were allowed free access to water but were fasted for 12 h before the experiment. Twelve rats were divided into two groups randomly (R-SC and P-SC groups). The rats were orally administered 18.0 g/kg R-SC or P-SC extract. Through an operation in the neck of the rat, a catheter was inserted to the jugular vein of the rat, thus the automated blood sampler could collect blood from the rat. The rat could free to move and kept awake. Blood samples (approximately 300 μL) were collected in heparinized tubes from each rat before and after oral administration at 0.083, 0.167, 0.25, 0.333, 0.5, 0.75, 1, 2, 4, 6, 8 and 12 h, using an automated blood sampler. The lost blood volume during collection was replaced with normal saline to prevent fluid loss from the animal.

Blood samples were centrifuged at 3000 rpm for 5 min, and 100 μL of plasma were finally obtained and stored at −80 °C until analysis.

### 4.9. Assay Application and Statistical Methods

All pharmacokinetic parameters were calculated using DAS 3.0 software (DAS 3.0, Mathematical Pharmacology Professional Committee of China, Shanghai, China). And the pharmacokinetic parameters were performed a non-compartmental analysis, including the maximum concentration (C_max_), the time to reach the maximum concentration (T_max_), area under the concentration–time curve (AUC_0-t_ and AUC_0-∞_) and mean residence time (MRT_0-t_). Each value is expressed as the mean ± SD.

All the data were checked and all of them were of normal distribution. And the analyses were performed using a *t*-test with SPSS 21.0 (SPSS Inc., Chicago, IL, USA). Values were considered significantly different at *p* < 0.05.

## 5. Conclusions

In this study, a sensitive, rapid and reliable UPLC–MS/MS method for the simultaneous quantification of seven anthraquinone aglycones in rat plasma has been established. This validated method was successfully used to evaluate the effect of processing on the pharmacokinetics of these anthraquinone aglycones in Semen cassiae. Our results indicated that as processing can increase C_max_ and AUC_0-12_, thus improving the bioavailability of the seven analytes. Moreover, processing can decrease the T_max_ of the seven analytes. This result explained to some extent that parching is required for this herbal medicine before clinical use.

## Figures and Tables

**Table 1:** The regression equations, linear ranges, LLOQs for the seven analytes in rat plasma.

Compounds	Regression Equation	r	Linear Range (ng/mL)	LLOQ (ng/mL)
aurantio-obtusin	*y* = 0.02099*x* + 0.00298	0.9976	0.94–564.0	0.94
obtusifolin	*y* = 0.04153*x* + 0.00092	0.9990	1.03–410.0	1.03
questin	*y* = 0.10900*x* + 0.00108	0.9997	0.84–84.0	0.84
SC-1	*y* = 0.04423*x* + 0.00072	0.9996	1.03–82.80	1.03
rhein	*y* = 0.00330*x* + 0.00147	0.9969	1.07–203.0	1.07
emodin	*y* = 0.05259*x* + 0.00383	0.9989	1.03–207.0	1.03
SC-2	*y* = 0.01045*x* + 0.00036	0.9964	0.90–180.0	0.90

**Table 2:** Precision and accuracy of the seven analytes in rat plasma (*n* = 3 days, 6 replicates per day).

Compounds	Concentration (ng/mL)	Intra-Day		Inter-Day	
		Accuracy (%)	RSD (%)	Accuracy (%)	RSD (%)
aurantio-obtusin	1.88	103.20	5.68	95.99	7.40
	18.8	99.86	2.72	103.38	5.66
	94	96.77	4.70	100.18	3.42
obtusifolin	2.05	96.76	6.96	103.27	6.97
	20.5	98.17	3.25	99.56	4.53
	103	100.37	2.65	103.90	7.32
questin	1.68	101.10	3.43	98.05	5.90
	16.8	99.99	4.91	103.51	4.83
	84	102.19	3.73	104.96	5.47
SC-1	2.07	96.29	4.65	101.74	5.33
	20.7	98.47	3.45	104.94	7.92
	103	99.93	4.98	97.02	4.01
rhein	2.13	104.69	5.25	103.45	7.74
	21.3	98.97	3.48	97.46	6.38
	107	101.57	3.83	102.89	4.68
emodin	2.07	105.60	4.96	104.16	3.97
	20.7	102.76	2.67	97.95	5.36
	103	104.62	4.82	95.52	8.65
SC-2	1.80	95.65	5.74	101.08	3.50
	18	98.65	3.56	97.13	3.16
	90	103.43	7.33	104.35	6.49

**Table 3:** Recovery and matrix effects for the seven analytes in rat plasma (*n* = 6).

Compounds	Concentration (ng/mL)	Recovery (%)		Matrix Effects (%)	
		Mean (%)	RSD (%)	Mean (%)	RSD (%)
aurantio-obtusin	1.88	91.13	5.68	98.51	4.55
	18.8	93.45	6.12	100.16	3.60
	94	89.80	5.71	97.06	5.58
obtusifolin	2.05	86.45	6.62	100.06	3.92
	20.5	88.25	7.56	94.46	4.30
	103	90.03	5.55	100.67	3.51
questin	1.68	86.72	4.84	95.40	5.22
	16.8	87.58	3.06	97.29	6.53
	84	89.76	2.45	93.50	6.29
SC-1	2.07	76.54	8.91	98.58	4.83
	20.7	78.90	4.87	96.77	3.24
	103	79.28	6.69	96.23	5.95
rhein	2.13	80.43	9.26	102.98	8.28
	21.3	81.72	5.65	99.27	3.28
	107	83.45	6.15	101.87	5.75
emodin	2.07	92.45	6.62	105.92	3.92
	20.7	93.77	5.61	103.07	3.54
	103	90.02	4.76	104.93	5.41
SC-2	1.80	93.23	5.85	97.94	6.63
	18	90.41	5.28	98.95	5.39
	90	87.64	7.50	99.74	4.41

**Table 4:** Stability of the seven analytes in rat plasma (*n* = 3).

Compounds	Concentration (ng/mL)	Room Temperature for 4 h		Autosampler for 24 h		−20 °C for 3 Weeks		Freeze-Thaw Cycles	
		Remains (%)	RSD (%)	Remains (%)	RSD (%)	Remains (%)	RSD (%)	Remains (%)	RSD (%)
aurantio-obtusin	1.88	103.7	4.62	104.8	5.54	104.49	5.13	106.89	8.40
	18.8	97.1	5.86	105.8	6.86	102.69	7.42	105.24	6.69
	94	95.4	7.65	97.8	4.58	97.84	5.17	98.05	4.43
obtusifolin	2.05	98.3	5.27	102.4	6.46	101.59	6.93	103.98	5.11
	20.5	91.5	5.93	93.4	7.39	93.69	6.73	95.74	5.81
	103	98.2	4.53	98.3	5.42	99.49	5.03	101.69	4.31
questin	1.68	99.0	4.03	100.8	4.72	98.14	5.42	103.45	3.78
	16.8	99.6	2.80	102.8	6.99	102.44	4.92	104.83	6.45
	84	105.5	6.37	99.1	4.40	103.54	2.41	96.70	5.09
SC-1	2.07	95.0	6.87	97.1	8.70	98.29	7.87	100.56	4.81
	20.7	97.9	4.06	99.4	4.76	99.89	4.46	102.14	7.81
	103	105.9	5.32	99.3	6.53	103.84	5.99	97.01	8.16
rhein	2.13	106.2	9.77	103.0	8.99	102.84	9.07	105.23	7.15
	21.3	94.9	4.47	95.9	5.30	96.64	4.93	98.77	4.29
	107	102.6	4.95	102.7	6.01	103.89	5.54	106.25	7.76
emodin	2.07	95.8	5.27	106.8	6.46	102.54	6.93	101.15	4.11
	20.7	99.3	4.58	102.2	5.48	101.99	5.08	96.36	4.36
	103	104.5	7.59	104.0	6.69	103.49	6.67	105.71	6.23
SC-2	1.80	105.5	4.74	93.5	5.71	100.74	5.28	95.65	4.53
	18	94.9	6.34	101.2	5.14	99.29	4.79	101.66	4.10
	90	105.5	5.96	103.6	7.43	106.29	6.77	97.54	5.81

**Table 5:** Pharmacokinetic parameters of the seven analytes after oral administration of R-SC and P-SC extract (*n* = 6, mean ± SD).

Compounds	Group	C_max_ (ng/mL)	T_max_ (h)	AUC_0-12_ (ng·h/mL)	AUC_0-∞_ (ng·h/mL)	MRT_0-12_ (h)
aurantio-obtusin	raw	171.46 ± 58.87	0.20 ± 0.07	537.31 ± 141.20	1436.22 ± 375.33	5.42 ± 1.19
	processed	438.36 ± 99.64 *	0.08 ±0.00 *	674.17 ± 87.38	846.48 ± 96.18	4.20 ± 0.54
obtusifolin	raw	99.13 ± 30.78	0.23 ± 0.07	279.63 ± 56.93	389.67 ± 58.93	5.17 ± 0.75
	processed	231.72 ± 39.96 *	0.13 ± 0.08	344.76 ± 44.90	385.30 ± 65.59	3.94 ± 0.40 *
questin	raw	28.35 ± 5.78	0.23 ± 0.07	110.56 ± 40.65	258.79 ± 45.11	5.45 ± 0.50
	processed	56.07 ± 8.05 *	0.17 ± 0.08	139.99 ± 30.00	218.56 ± 42.14	4.83 ± 0.48
SC-1	raw	11.34 ± 2.65	0.17 ± 0.00	60.60 ± 10.47	152.07 ± 43.76	6.49 ± 1.11
	processed	28.74 ± 6.55 *	0.08 ± 0.00 *	70.53 ± 15.62	145.87 ± 36.46	6.25 ± 0.62
rhein	raw	39.48 ± 6.72	0.23 ± 0.07	155.19 ± 32.53	351.71 ± 54.77	5.45 ± 0.54
	processed	75.99 ± 9.68 *	0.13 ± 0.07	192.33 ± 26.31	294.23 ± 45.80	3.74 ± 0.42 *
emodin	raw	4.94 ± 1.40	0.18 ± 0.04	33.67 ± 7.85	80.72 ± 21.59	7.15 ± 0.74
	processed	9.75 ± 1.94	0.17 ± 0.06	38.43 ± 8.13	82.66 ± 19.56	6.24 ± 0.50
SC-2	raw	4.07 ± 1.36	0.10 ± 0.04	21.08 ± 4.37	70.47 ± 13.66	6.40 ± 0.43
	processed	9.64 ± 1.75	0.12 ± 0.08	25.12 ± 5.79	48.10 ± 9.22	6.29 ± 0.48

* *p* < 0.05.

**Table 6:** The dosages of these analytes in the R-SC and P-SC group.

Compounds	Dosages in R-SC Group (mg/kg)	Dosages in P-SC Group (mg/kg)
aurantio-obtusin	5.78	9.18
obtusifolin	1.78	3.52
questin	0.28	0.40
SC-1	0.96	1.63
rhein	0.26	0.40
emodin	0.74	1.17
SC-2	0.44	0.76
